# Predicting the unseen: nutritional interventions as a key to combat frailty

**DOI:** 10.3389/fnut.2025.1575922

**Published:** 2025-07-09

**Authors:** Luya Shi, Xijiang Tian, Yih Bongsook, Jiameng Chen

**Affiliations:** ^1^Department of Nursing, Municipal Hospital Affiliated to Taizhou University, Taizhou, Zhejiang, China; ^2^Post Graduate School of Nursing, Sehan University, Yeonggam, Republic of Korea; ^3^Weifang University of Science and Technology, Weifang, Shandong, China; ^4^Department of Nursing, Taizhou Central Hospital (Taizhou University Hospital), Taizhou, Zhejiang, China

**Keywords:** bibliometric analysis, Bibliometrix, Citespace, frailty, nutrition status, elderly, VOSviewer

## Abstract

**Background:**

Frailty and malnutrition have emerged as critical public health issues amidst global population aging. Malnutrition not only significantly contributes to frailty but also intensifies its clinical symptoms, severely affecting the quality of life and health outcomes in older adults. Research in this field has accelerated in recent years; however, a comprehensive analysis of key research trends and hotspots remains absent. This study employs bibliometric methods to systematically analyze core themes and emerging research directions related to nutritional status and frailty in older adults, identifying potential research frontiers and guiding future development.

**Methods:**

A comprehensive search was conducted in the Web of Science Core Collection (WoSCC) database on November 6, 2024, using keywords relevant to frailty and nutrition status in older adults. Bibliometric analyses and knowledge mapping were performed using CiteSpace, VOSviewer, and R software.

**Results:**

Between 2005 and 2024, 2,357 publications on frailty and nutrition status in older adults were produced by 13,080 researchers from 3,987 institutions across 88 countries. The volume of publications has shown a consistent upward trajectory over the past two decades (R^2^ = 0.84), with projections indicating a continued increase, peaking at 315 publications by 2033. This sustained growth underscores the field’s significance and ongoing research interest. Early research has centered on the “home-living elderly” demographic, while current investigations have shifted focus from molecular biology, genetics, and health nursing to more clinical and medical domains. Key areas of emphasis now include nutrition and dietetics, geriatrics, oncology, and pharmacology. Emerging research hotspots involve the early identification and management of malnutrition to reduce frailty-related health risks and improve health outcomes and quality of life for older adults. Notable trends include the keywords “prediction,” “nutritional assessment MNA,” “intervention,” and “infection.”

**Conclusion:**

This bibliometric analysis offers a comprehensive examination of the research evolution, hotspots, and emerging frontiers in frailty and nutrition status among older adults over the past two decades. The findings provide an objective overview of the academic landscape, offering valuable insights for future research, resource allocation, and policymaking.

## 1 Introduction

Frailty has become a major global health challenge, driven by the rapid acceleration of population aging. It is a prevalent and severe geriatric syndrome marked by diminished physiological reserves and multi-system dysfunction, which reduces the ability to adapt to stressors and significantly elevates the risk of adverse outcomes, including functional decline, falls, hospitalization, and mortality in older adults (1). The prevalence of frailty among older adults is estimated at 24.0%, rising to 51.5% in nursing home residents and those with chronic diseases (2–4). It disproportionately affects women and worsens with advancing age (2, 3, 5). As aging populations grow, frailty prevalence is projected to follow an upward trajectory. Studies report that frailty increases the risk of premature mortality by 1.8–2.3 times, falls and fractures by 1.2–2.8 times, hospitalization by 1.2–1.8 times, and functional impairment in activities of daily living by 1.6–2.0 times (6). Beyond its health implications, frailty imposes significant quality-of-life reductions for older adults while creating substantial economic and caregiving burdens for families and society (7, 8). Consequently, effective frailty prevention and management have emerged as research priorities in aging societies.

Nutrition status is a pivotal modifiable factor and a key intervention target for the prevention and management of frailty (9–11). Evidence indicates that poor nutritional status not only heightens frailty risk but also accelerates its progression (10–12). Furthermore, it is strongly associated with an increased all-cause mortality rate over an 8-year period among older adults (13, 14). Frail individuals are five times more likely to experience malnutrition than their non-frail counterparts, and addressing malnutrition could potentially prevent 2.5%–5.0% of frailty cases (15). Early screening and intervention for malnutrition are thus critical components of frailty management strategies. Research suggests that the interplay between malnutrition and frailty operates through mechanisms such as micronutrient deficiencies, cognitive impairment, physical weakness, and reduced quality of life (16–20). Widely used nutrition assessment tools for older adults include the Mini Nutritional Assessment (MNA), Subjective Global Assessment (SGA), and Nutritional Risk Screening-2002 (NRS-2002) (21). Key intervention strategies involve dietary supplementation (particularly protein), combined nutrition and physical activity programs, personalized nutritional counseling, and multimodal approaches incorporating social support (22). Over the past two decades, research on the relationship between nutritional status and frailty in older adults has expanded exponentially, encompassing epidemiological studies, investigations into pathological mechanisms, intervention strategies, and prognostic evaluations. However, systematic analyses of the current research landscape, emerging trends, and research hotspots in this field remain scarce, highlighting the need for further exploration.

Bibliometrics provides a robust framework for quantitatively analyzing academic literature, leveraging mathematical and statistical methods to evaluate the relationships and impact of publications, authors, institutions, and countries within a given research domain (23). This approach uncovers the developmental trajectory and emerging trends of a field, offering data-driven insights and a theoretical basis for future investigations (23). In recent years, bibliometric analysis has been extensively utilized in medical research, encompassing areas such as the molecular mechanisms underlying frailty, frailty biomarkers, its relationship with falls, and the effectiveness of exercise interventions (24–26). Despite its broad application, no bibliometric studies have specifically addressed the intersection of nutrition status and frailty. This study employs bibliometric methods to systematically examine the research landscape, key trends, and developmental patterns in the field of nutritional status and frailty among older adults. By identifying research hotspots and emerging dynamics, it seeks to establish a theoretical framework and practical references to inform academic inquiry, optimize resource allocation, and guide policy development in this area. The findings aim to advance the exploration of effective strategies for frailty prevention and management, ultimately enhancing health outcomes and quality of life for older adults.

## 2 Materials and methods

### 2.1 Data collection

**FIGURE 1:**
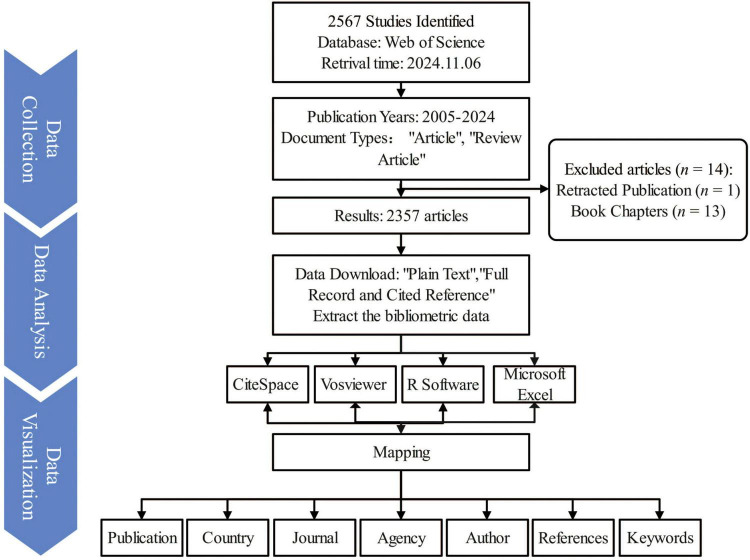
Overview of the research design and literature screening process in the bibliometric study of nutritional status and frailty in older adults. The flowchart illustrates the data source, inclusion and exclusion criteria, final dataset selection, and key phases of bibliometric analysis, including co-authorship, keyword clustering, and co-citation analysis. This figure visually summarizes the methodological framework and analytical workflow used in the study.

The Web of Science Core Collection (WoSCC) database is renowned for its comprehensive coverage of high-impact academic literature globally, making it a key resource for bibliometric analysis. In this study, the WoSCC database was utilized to perform a bibliometric analysis of publications related to frailty and nutritional status in older adults. To ensure thorough and precise retrieval, relevant keywords were compiled from previous literature reviews and incorporated MeSH terms from PubMed to finalize the search query. On November 6, 2024, a targeted search was executed in WoSCC using the following search string in the WOSCC database: TS = (Nutrition* Status) AND TS = (“Debilit*” OR “Frail*” OR “Astheni*” OR “Lassitud*” OR “fatigu*”) AND TS = (senior* or elder* or old* or aged or aging or postmenopaus* or geriatric*). This search retrieved 2,567 publications. Document type filters were applied (limiting results to articles and reviews), with exclusions for retracted publications and book chapters, and the publication period restricted to 2005–2024, resulting in a refined dataset of 2,357 publications (Figure 1). To maintain data accuracy, all searches, data extraction, and downloads were independently conducted by two researchers on the same day, with any discrepancies resolved through consultation with a third researcher.

### 2.2 Data analysis

CiteSpace was utilized to eliminate duplicate publications, merge keywords, and facilitate other data preprocessing tasks. Subsequent analysis was performed using VOSviewer 1.6.20, CiteSpace 6.3.R1, Bibliometrix, R software, and Microsoft Excel 2019.

#### 2.2.1 CiteSpace

CiteSpace, a Java-based bibliometric and visual analysis software, was employed to examine structural indicators (e.g., betweenness centrality), temporal indicators (e.g., citation bursts, timelines, landscapes, time zones), and the interrelationships among clusters (e.g., cluster dependency) across countries, institutions, authors, journals, keywords, and co-cited literature (27–29). In the generated visualization, the thickness of connecting lines indicates relationship strength, while node size corresponds to the frequency of appearance. Software parameters were configured with a Time Slice of 1 year per slice, and the Selection Criteria was set to g-index: *k* = 25.

#### 2.2.2 VOSviewer

VOSviewer, a widely recognized tool in bibliometric analysis, was utilized to construct networks of author collaboration and keyword co-occurrence (30). These relationships are visualized as nodes and lines of varying colors and sizes, representing the connections and frequencies of each element’s occurrence within the dataset.

## 3 Results

### 3.1 Annual publications and trend

**FIGURE 2:**
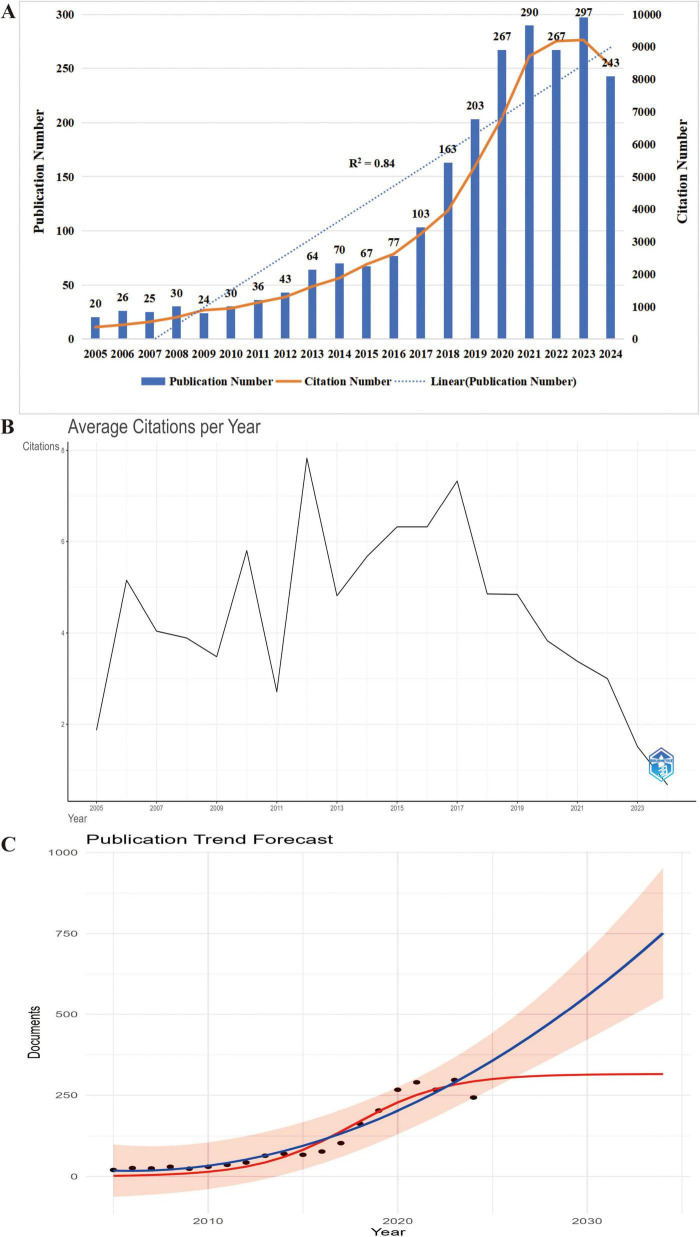
Temporal trends and future projection of research output in nutritional status and frailty among older adults. **(A)** Annual number of publications (bars) and citations (line) from 2005 to 2024, highlighting steady growth in scholarly attention to this field. **(B)** Average annual citation count by publication year, indicating the long-term academic influence of earlier studies (2005–2024). The *x*-axis represents the year of publication, with all subsequent citations accumulating in the respective year. **(C)** Trend prediction of annual publications on nutritional status and frailty in older adults (2024–2034). Black dots represent actual publication counts from 2005 to 2024, while the red curve illustrates the projected annual publication trend. The prediction indicates a steady increase, suggesting sustained and expanding research interest in this field.

Analyzing publication volume and trends offers valuable insights into the academic significance and global research activity of a field (28). On November 6, 2024, a total of 2,357 publications on frailty and nutrition status in older adults were retrieved from the WoSCC database, accumulating 62,583 citations, with an average of 26.69 citations per publication, an H-index of 110, and an average document age of 4.9 years. The average document age was calculated as the mean difference between the year of data retrieval (2024) and the publication year of each included document. The 2024 data reflect publications indexed up to November 6, representing partial-year counts without extrapolation. From 2005 to 2024, the field exhibited rapid growth in annual publication volume (Figure 2A). In 2023, 297 papers were published—almost 15 times the 20 papers published in 2005. A significant increase in publication volume began in 2013, exhibiting a strong linear growth trend (R^2^ = 0.84). Notable publication peaks occurred in 2014, 2021, and 2023. The total number of citations also increased steadily, with 9,210 citations recorded in 2023, the highest for any year. Citation analysis (Figure 2B) highlighted influential articles published in 2006, 2010, 2012, and 2017, which garnered higher citation counts. Future publication trends (Figure 2C) predict continued growth, with 292 papers expected in 2024 and a projected peak of 315 papers around 2033. These trends underscore the sustained academic interest and growing significance of research on frailty and nutritional status among older adults, signaling continued engagement from the research community in the years ahead.

### 3.2 Analysis of countries

**FIGURE 3:**
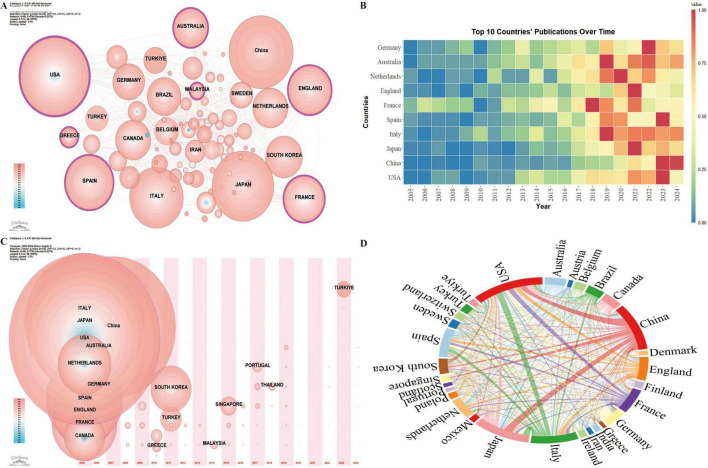
Geospatial analysis of research output on nutritional status and frailty in older adults. **(A)** Co-occurrence network of contributing countries. Node size corresponds to the frequency of co-occurrence, while nodes encircled in purple indicate high centrality (≥0.1), signifying influential bridging roles. **(B)** Temporal trends in annual publication output from the top 10 most prolific countries. **(C)** Co-occurrence network of contributing countries by publication timezone. The horizontal axis displays the year of each country’s initial publication in frailty and nutritional status research. Node size represents the publication volume, with blue nodes indicating earlier contributions and pink nodes signifying recent publications. Overlapping colors indicate multiple publications within a single year, forming rings that reflect consistent and extensive publication activity over time, demonstrating each country’s ongoing engagement in the field. **(D)** International collaboration network with link thickness representing the strength of inter-country collaboration.

Country-level analysis highlights the most influential nations in the field of frailty and nutrition status in older adults, as well as patterns of international collaboration. A total of 88 countries have contributed research in this area, with seven countries exhibiting high betweenness centrality (Figure 3A): the USA (0.23), England (0.18), France (0.17), Australia (0.17), Spain (0.16), Malaysia (0.13), and Greece (0.11). Betweenness centrality reflects the critical role these countries play in connecting various parts of the research network. Among the top ten countries by publication volume (Figure 3B), the USA (364), China (305), and Japan (294) are the leading contributors. In terms of H-index, the USA (65), Italy (54), and France (39) are at the forefront. Analysis of the first publication year and annual publication trends (Figure 3C) indicates that the leading countries began publishing in this field between 2005 and 2006 or earlier.

International collaboration analysis among the 30 countries with over 20 publications reveals strong research partnerships, particularly between China, the USA, Japan, and Italy (Figure 3D).

### 3.3 Analysis of institutions

**FIGURE 4:**
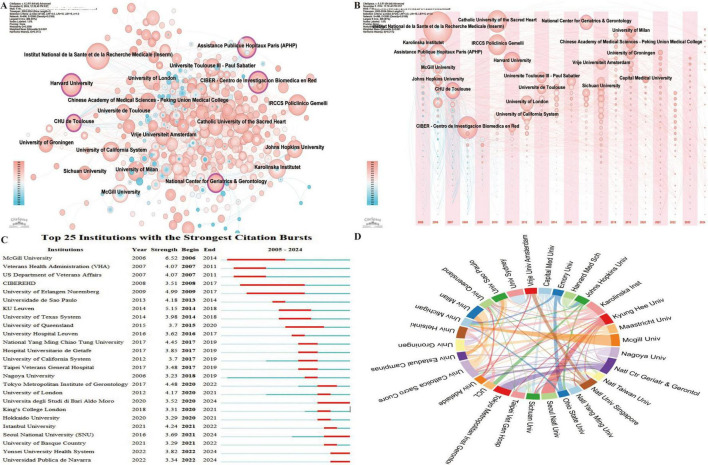
Institutional collaboration network in research on nutritional status and frailty in older adults. **(A)** Co-occurrence network of contributing institutions. Node size corresponds to the frequency of co-occurrence, while nodes encircled in purple indicate high centrality (≥0.1), signifying influential bridging roles. **(B)** Co-occurrence network showing the timezone of contributing institutions. **(C)** Top 25 institutions with the strongest citation bursts, highlighting periods of notable academic impact. **(D)** Collaboration network among institutions, where link thickness indicates the strength of collaboration.

Institutional analysis identifies the leading institutions in the field and examines their collaborative dynamics, offering valuable insights for future academic exchanges. A total of 3,987 institutions have contributed research on frailty and nutrition status in older adults. Among them, five institutions demonstrate high betweenness centrality (Figure 4A): Harvard University (0.18), Assistance Publique Hôpitaux Paris (APHP) (0.12), the National Center for Geriatrics & Gerontology (0.11), CIBER - Centro de Investigación Biomédica en Red (0.10), and CHU de Toulouse (0.10), underscoring their central roles in the research network.

Analysis of the first publication year and subsequent annual output (Figure 4B) reveals that the top ten institutions began publishing in this field between 2005 and 2007. The institutions with the highest publication volumes include France’s Institut National de la Santé et de la Recherche Médicale (INSERM) (72), Spain’s Centro de Investigación Biomédica en Red (CIBER) (48), and Italy’s Catholic University of the Sacred Heart (47). Leading institutions by H-index include INSERM (27), Catholic University of the Sacred Heart (26), and IRCCS Policlinico Gemelli (26).

Regarding citation bursts, which indicate periods of rapid citation increase reflecting emerging influence, McGill University recorded the earliest and most intense burst in 2006 (strength = 6.52) (Figure 4C), while CIBER-EHD experienced the longest burst from 2008 to 2017. Institutions such as Yonsei University Health System and Universidad Pública de Navarra continue to experience citation bursts.

Collaboration analysis among the 30 institutions with over 15 publications (Figure 4D) reveals strong partnerships, notably between McGill University and the University of Michigan (USA), Seoul National University and Kyung Hee University (Korea), and the National Center for Geriatrics & Gerontology and Tokyo Metropolitan Institute of Gerontology (Japan). The international co-authorship rate stands at 20.38%, highlighting the need for further global collaborative nature of this research.

### 3.4 Analysis of authors

**FIGURE 5:**
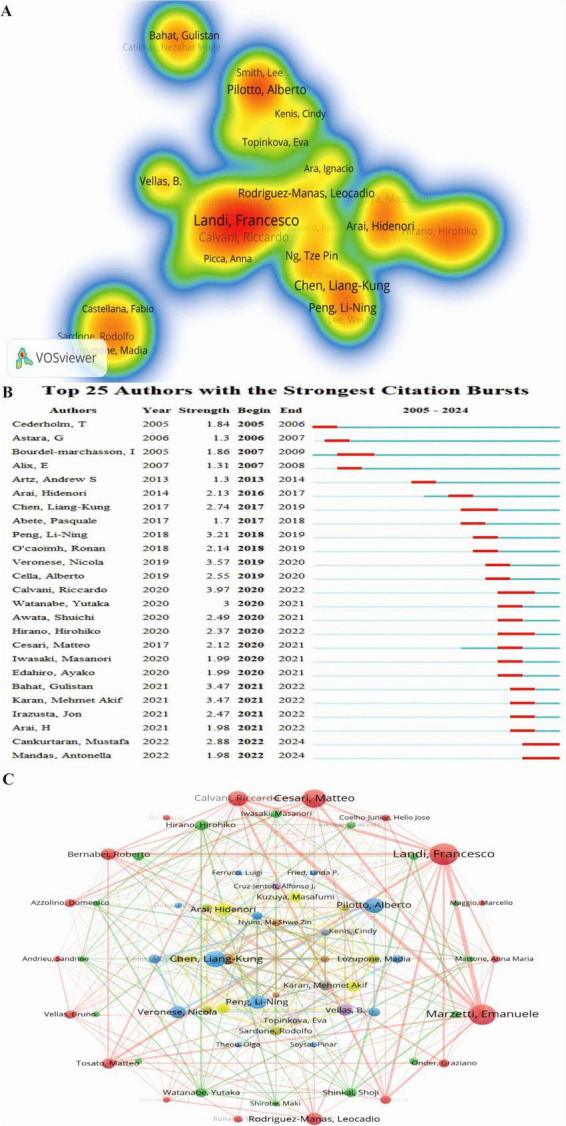
Author collaboration network in research on nutritional status and frailty in older adults. **(A)** Author co-occurrence heatmap. The intensity of color represents the frequency of co-occurrence between authors, with red color indicating higher co-occurrence frequencies. **(B)** Top 25 authors with the strongest citation bursts, highlighting periods of intense academic influence. **(C)** Co-authorship network of contributing authors in nutritional status and frailty among older adults. Node size reflects co-authorship frequency, and connecting lines represent shared publications, revealing key scholarly networks in the field.

Author analysis identifies leading experts and core research teams, offering valuable insights for tracking research trends and fostering collaboration. A total of 13,080 authors have contributed to research on frailty and nutritional status in older adults. Among them, the top three authors by both publication volume and H-index are Vellas Bruno, De Vito Francesco, and Marzetti Emanuele (Figure 5A). The Top 25 Authors with the Strongest Citation Bursts (Figure 5B) shows that Calvani Riccardo has the most significant citation burst (strength = 3.97), with Cankurtaran Mustafa and Mandas Antonella continuing to experience citation bursts.

Using VOSviewer, collaborations among the 141 authors with more than five publications were analyzed (Figure 5C), revealing 12 clusters based on research focus and collaboration patterns. The largest cluster (outermost red layer) consists of 18 authors, with Landi Francesco, Marzetti Emanuele, and Cesari Matteo at its core. The second-largest cluster (green, second layer) includes 16 authors, with Hirano Hirohiko, Shinkai Shoji, and Watanabe Yutaka as central figures. The third-largest cluster (blue, third layer) consists of 10 authors, with Pilotto Alberto, Veronese Nicola, and Cella Alberto at its center. Collaboration spans all 12 clusters, reflecting the interconnected nature of research in this field.

### 3.5 Analysis of journals

**FIGURE 6:**
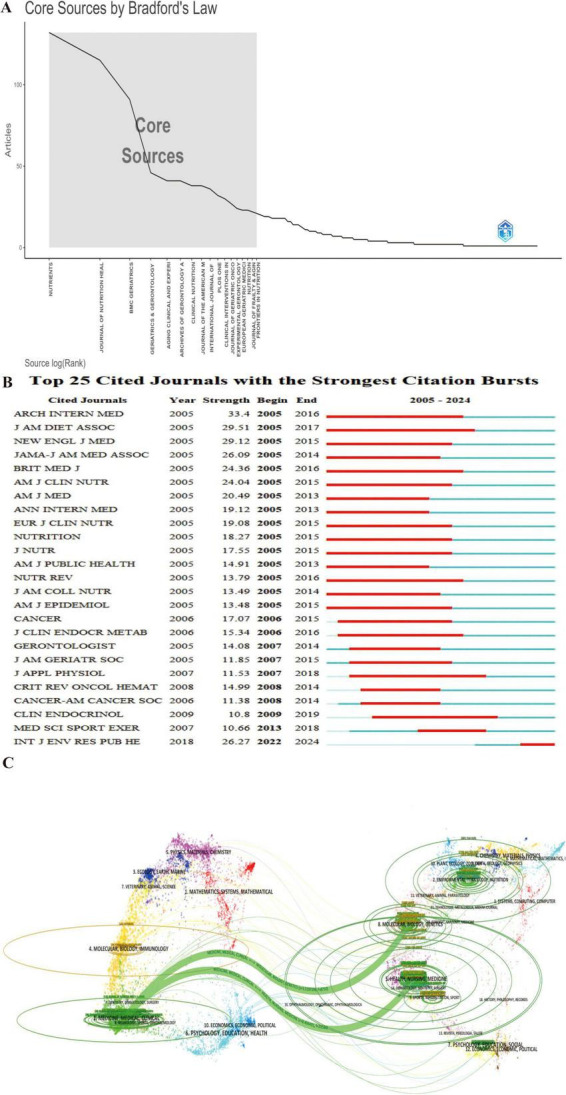
Journal-level analysis of publication and citation trends in frailty and nutrition research. **(A)** Bradford’s Law distribution identifying core journals. **(B)** Top 25 cited journals with the strongest citation bursts, demonstrating evolving journal-level impact. **(C)** Dual-map overlay showing citation flows between citing (left) and cited (right) journal clusters. Arrows indicate cross-disciplinary knowledge transfer between research domains.

Journal analysis provides insights into impact, trends, and research priorities, guiding researchers in journal selection and identifying emerging topics. A total of 785 journals have published studies on frailty and nutrition status among older adults. Bradford’s Law (also called Bradford’s Law of Scattering) describes the distribution of scientific literature across journals. Journals can be divided into zones based on productivity: a small core zone with the most articles, followed by zones with progressively larger numbers of less productive journals. Following Bradford’s Law of Core Sources (Figure 6A), the top ten journals by publication volume and H-index are *Nutrients, Journal of Nutrition Health & Aging, BMC Geriatrics, Geriatrics Gerontology International, Aging Clinical and Experimental Research, Archives of Gerontology and Geriatrics, Clinical Nutrition, Journal of The American Medical Directors Association, International Journal of Environmental Research and Public Health*, and *PLos One*. These journals account for 26.14% of total publications (2,357).

The Top 25 Cited Journals with the Strongest Citation Bursts (Figure 6B) reveals that the *International Journal of Environmental Research and Public Health* is currently in a burst phase, indicating growing influence. *Journal of the American Dietetic Association* experienced the longest burst duration (2005–2017), while *Archives of Internal Medicine* saw the most intense burst (strength = 33.4).

The dual-map overlay of journals (Figure 6C) illustrates the development and trends in the field. Citing journals on the left primarily belong to the Medicine, Medical, and Clinical domain, while cited journals on the right cover Molecular Biology, Genetics, Health, Nursing, and Medicine. The colorful tracks connecting the citing and cited fields highlight the flow of knowledge, with thicker tracks indicating higher citation frequencies. This visualization indicates a shift in the field of frailty and nutrition status research, from an emphasis on Molecular Biology and Genetics toward Medicine and Clinical Science.

### 3.6 Analysis of references

Reference analysis is a key method for identifying the knowledge structure, research hotspots, interdisciplinary connections, and academic influence within a field. It enables researchers to assess the historical and current landscape of a domain, providing a solid foundation for future research directions and strategic decisions (27). An analysis of the top 10 most-cited articles in this area (31–40) (Supplementary Table 1) revealed four reviews and six original research articles, with citation counts reaching as high as 2,573. These studies explore the relationship between frailty and nutrition status in older adults, focusing on areas such as gut health, dysphagia, nutritional assessment, and personalized interventions. Collectively, they emphasize that early identification of frailty, risk factor evaluation, and diversified intervention strategies can significantly enhance nutritional status, reduce frailty risk, and improve overall quality of life.

**FIGURE 7:**
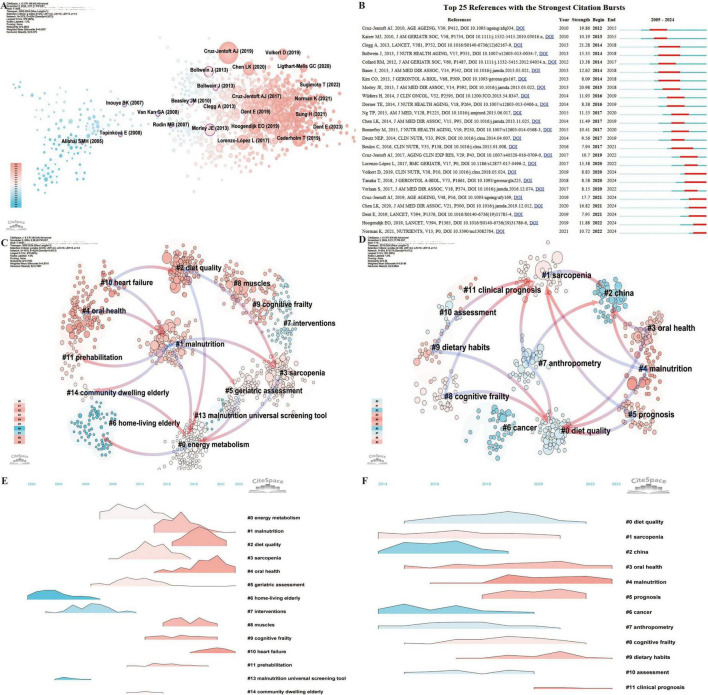
Co-citation and clustering analysis of references reveals major thematic domains and their temporal evolution. **(A)** Co-citation network visualization from 2005 to 2024, where node size represents citation frequency. The transition from pink to blue in node color reflects the temporal distribution of publications, with pink denoting more recent works. **(B)** Top 25 references with the strongest citation bursts from 2005 to 2024, highlighting influential works during key timeframes. **(C)** Reference clustering based on semantic similarity from 2005 to 2024, with pink arrows illustrating the primary flow of citations within each cluster. **(D)** Reference clustering based on semantic similarity from 2019 to 2024, capturing recent topic structures. **(E)** Landscape map of reference clusters from 2005 to 2024, showing spatial relationships among thematic areas. **(F)** Landscape map of reference clusters from 2019 to 2024.

Using CiteSpace to conduct a betweenness centrality analysis of references, 10 high-centrality articles were identified (Figure 7A) (41–50). These articles address diverse topics, including nutrient intake, frailty assessment, weight management, and dietary quality. They underscore the critical role of nutrition in managing frailty, demonstrating that scientific assessments, targeted dietary interventions, and multidisciplinary approaches can mitigate physical and psychological decline, improve the quality of life, and promote better health outcomes for older adults.

The Top 25 References with the Strongest Citation Bursts (Figure 7B) reveal that the 2013 review by Clegg et al. published in The *Lancet* titled “Frailty in elderly people” (51), experienced the longest and most intense citation burst (2014–2018; Strength = 21.28). This influential work highlighted frailty as a major challenge linked to population aging and associated with adverse health outcomes. It also called for improved detection methods to enable more effective, targeted care. Currently, seven references remain in the burst phase (52–58), covering topics such as nutritional assessment, frailty risk factors, sarcopenia diagnosis, and the interplay between malnutrition and frailty. Collectively, these studies stress the critical importance of early identification and management of malnutrition to reduce frailty progression, mitigate health risks, and improve quality of life for older adults, making this area a prominent research hotspot.

Reference clustering analysis offers further insight, revealing not only current research hotspots but also the evolution of the field over time (Figure 7C). The arrows in the timeline highlight the relationships between clusters, with pink arrows representing foundational knowledge and blue arrows indicating emerging frontiers. Cluster #6 (home-living elderly) represents the foundational research on frailty and nutrition status in older adults. This foundational work subsequently expanded into Cluster #0 (energy metabolism) and later evolved into Cluster #14 (community-dwelling elderly), Cluster #5 (geriatric assessment), Cluster #3 (sarcopenia), and Cluster #2 (diet quality). The most recent research directions are captured in Cluster #4 (malnutrition), Cluster #3 (oral health), Cluster #5 (prognosis), Cluster #9 (dietary habits), and Cluster #8 (cognitive frailty) (Figure 7D). The reference clustering landscape (Figures 7E, F) underscores the rapid growth of Cluster #3 (oral health) and Cluster #4 (malnutrition) in recent years, marking them as current research hotspots. This dynamic analysis highlights the evolving nature of the field, with a growing focus on addressing critical issues like malnutrition, oral health, and their impact on frailty, prognosis, and the overall quality of life for older adults.

### 3.7 Hotspots and frontiers

**FIGURE 8:**
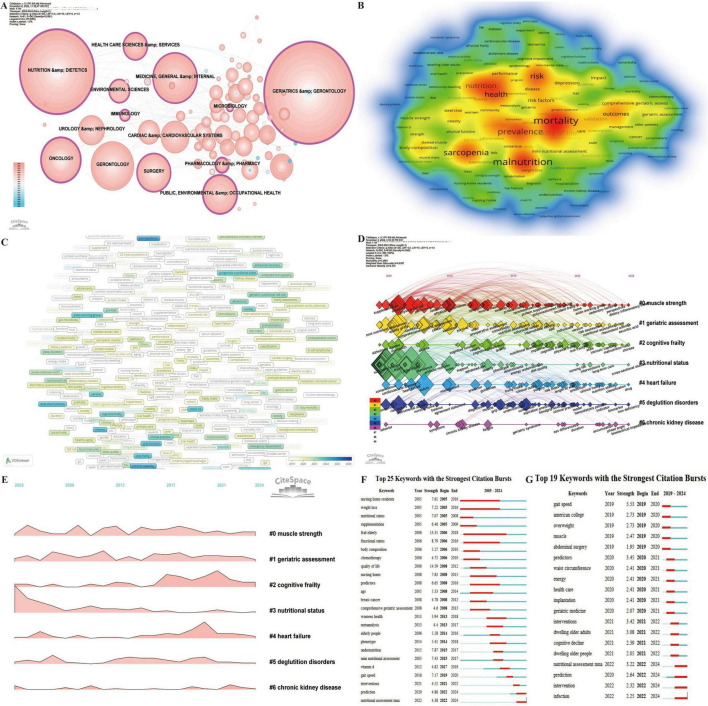
Analytical overview of research domains and key terms in the study of nutritional status and frailty in older adults. **(A)** Co-occurrence network of research fields and disciplines, where node size reflects citation frequency. **(B)** Keyword co-occurrence heatmap. The intensity of color represents the frequency of co-occurrence between keywords, with red color indicating higher co-occurrence frequencies. **(C)** Co-occurrence network of prominent keywords from 2019 to 2024, highlighting emerging research focuses. **(D)** Timeline map of keyword clusters from 2005 to 2024, showing the evolution of research topics over time. **(E)** Landscape map of keyword clusters from 2005 to 2024. **(F)** Top 25 keywords with the strongest citation bursts from 2005 to 2024, capturing shifts in thematic emphasis. **(G)** Top 25 keywords with the strongest citation bursts from 2019 to 2024.

Keyword analysis is a pivotal metric in bibliometric studies, offering insights into the evolution of research fields, developmental trends, and the identification of emerging hotspots (28, 59). Through disciplinary analysis of keywords (Figure 8A), 11 disciplines with high betweenness centrality were identified, including public, environmental and occupational health (0.28), nutrition and dietetics (0.23), geriatrics and gerontology (0.22), oncology (0.20), pharmacology and pharmacy (0.20), surgery (0.15), health care sciences and services (0.15), immunology (0.13), medicine, general and internal (0.12), environmental sciences (0.12), and microbiology (0.12). These results underscore the interdisciplinary nature of research on frailty and nutrition status in older adults while highlighting the key focus areas within the field.

Using VOSviewer for co-occurrence analysis of keywords (Figure 8B), the top 20 most frequent terms, excluding “frailty,” “elderly,” and “nutrition status,” were identified. These include mortality (512), malnutrition (500), prevalence (405), sarcopenia (401), risk (361), health (334), quality of life (324), association (269), disability (203), outcomes (203), validation (170), risk factors (160), depression (155), impact (144), index (135), body-composition (134), exercise (132), comprehensive geriatric assessment (120), care (114), and survival (113). These keywords reflect a comprehensive focus on the relationships between malnutrition and frailty in older adults, along with their effects on brain health outcomes, assessment tools, interventions, and management strategies. This highlights the complex, multifaceted nature of frailty and nutrition-related issues in aging populations. Effective interventions that target malnutrition risk, early detection, and physical and mental health support can significantly improve outcomes, quality of life, and longevity in frail older adults.

Analysis of the average appearance year (AAY) for 674 keywords appearing more than five times from 2019 to 2024 (Figure 8C) reveals emerging terms such as bioimpedance, fatty acids, intrinsic capacity, oral frailty, loneliness, malnutrition risk, and oral function, signaling these as current research hotspots.

Keyword clustering analysis (Figures 8D, E) further categorized keywords into seven clusters. Among these, Cluster #0 (muscle strength), Cluster #1 (geriatric assessment), and Cluster #2 (cognitive frailty) have exhibited rapid growth and remain highly active. Notably, the latest keywords for Cluster #0 (muscle strength) include sedentary behavior, perspectives, and dietary inflammatory index; for Cluster #1 (geriatric assessment) the new keywords are nutritional assessment MNA, rheumatoid arthritis, and ascorbic acid; while Cluster #2 (cognitive frailty) includes intrinsic capacity, malnutrition risk, and Chinese.

Keyword citation bursts provide a critical metric for identifying temporal shifts in research priorities and emerging trends, enabling a dynamic understanding of evolving focus areas within a field. Analysis of the Top 25 Keywords with the Strongest Citation Bursts (2005–2024) (Figure 8F) revealed that “prediction” and “nutritional assessment MNA” remain active bursts, while the longest-lasting burst was “frail elderly” (2006–2018), and the strongest burst was “quality of life” (Strength = 14.59). A focused analysis of the Top 19 Keywords with the Strongest Citation Bursts (2019–2024) (Figure 8G) highlights ongoing trends in nutritional assessment MNA, prediction, intervention, and infection.

The temporal evolution of keywords illustrates the progressive development of this research domain (Figure 8F). Early studies prioritized environmental factors and frailty risk, emphasizing the effects of weight loss, body composition, functional status, and nutritional status among nursing home residents. Prominent keywords during this period included nursing home residents, weight loss, nutritional status, supplementation, frail elderly, body composition, and functional status. The focus later shifted to disease-related malnutrition, particularly addressing health concerns in older women, such as the nutritional and quality-of-life impact of breast cancer chemotherapy. Keywords like chemotherapy, quality of life, nursing home, predictors, age, breast cancer, and women’s health characterized this stage. Recent research has concentrated on nutritional screening, prediction, and intervention efficacy. This phase emphasizes early identification of malnutrition using predictive models and tools such as the MNA, alongside nutritional supplementation and multi-intervention strategies aimed at improving nutritional status, preventing frailty progression, and enhancing health outcomes and quality of life in older adults. Emerging keywords include meta-analysis, elderly people, phenotype, undernutrition, MNA, vitamin D, gait speed, interventions, prediction, and nutritional assessment MNA. This analysis underscores the dynamic and evolving nature of research on frailty and nutritional status among older adults, highlighting its increasing relevance in addressing aging-related health challenges and improving outcomes in this population.

## 4 Discussion

### 4.1 General analysis

Frailty and malnutrition have emerged as critical public health challenges in aging societies worldwide. Malnutrition, a significant risk factor for frailty, exacerbates its symptoms, negatively impacting the quality of life and health outcomes of older adults (10–12, 16–19). Early screening and nutritional interventions are therefore essential in preventing and delaying frailty while enhancing the overall well-being of older populations. This study employs bibliometric analysis to examine 2,357 publications on frailty and nutrition status in older adults indexed in the WOSCC database from 2005 to 2024. Results indicate a linear increase in annual publications over the past two decades (R^2^ = 0.84), with projections suggesting a peak in publication volume at approximately 315 papers by 2033. This trend underscores the growing importance of this research area and its potential to attract greater attention from the scientific community in the coming years.

From the perspective of research contributions, countries with high betweenness centrality include the USA, England, France, Australia, Spain, Malaysia, and Greece (Figure 3A). The USA, China, and Japan lead in publication volume, while the USA, Italy, and France rank highest in H-index scores. These findings highlight the influential roles of the USA and France in this field, with China and Japan’s research impact requiring further development. The prominence of the USA is attributed to its leading research institutions, well-funded interdisciplinary studies, and advanced healthcare system. France’s longstanding research tradition in geriatrics, public health, and nutrition, bolstered by renowned academic institutions focused on aging, also contributes significantly to the field. Both countries benefit from extensive health databases and longitudinal projects, providing invaluable data on the prevalence, risk factors, and progression of frailty and malnutrition (60).

At the institutional level, Harvard University, APHP, the National Center for Geriatrics & Gerontology, CIBER, and CHU de Toulouse exhibit high betweenness centrality (Figure 4A). INSERM, CIBER, and the Catholic University of the Sacred Heart are the top institutions in terms of publication volume, while INSERM, the Catholic University of the Sacred Heart, and IRCCS Policlinico Gemelli lead in H-index scores. These institutions are central to the field, yet collaboration remains predominantly domestic, suggesting an opportunity to strengthen international partnerships. Leading authors in this field include Vellas Bruno, De Vito Francesco, and Marzetti Emanuele, who rank highly in both publication volume and H-index scores (Figure 5A). Top journals in the field include *Nutrients*, *Journal of Nutrition Health & Aging*, and *BMC Geriatrics* (Figure 6A). A shift in focus from Molecular Biology, Genetics, and Health Nursing to Medicine and Clinical Research (Figure 6C) reflects the field’s transition from foundational research to clinical applications. Current studies increasingly aim to integrate molecular and genetic insights into personalized clinical treatment strategies. Researchers can monitor key countries, institutions, authors, and journals to track emerging research hotspots and advancements and identify potential collaboration opportunities.

Reference analysis is a vital tool for researchers, providing a detailed understanding of the historical and current state of a scholarly field while offering evidence-based guidance for future research priorities and strategic planning (27). The top 10 most-cited references (31–40) (Supplementary Table 1) focus primarily on the relationship between frailty and nutritional status, collectively highlighting the importance of early frailty detection, comprehensive risk factor assessment, and multifaceted interventions in enhancing nutritional status, reducing frailty risk, and improving the overall quality of life in older adults. The ten references with the highest betweenness centrality (Figure 7A) further emphasize the critical role of nutritional status and dietary quality in frailty management (41–50). These studies advocate for strategies such as frailty assessment, targeted dietary interventions, and multidisciplinary care to optimize health outcomes in frail older populations.

Analysis of the Top 25 References with the Strongest Citation Bursts (Figure 7B) identifies the 2013 review article “Frailty in Elderly People” by Clegg A. in The *Lancet* as having the longest and most intense citation burst (2014–2018, Strength = 21.28) (51). This landmark study underscores frailty as a critical challenge in aging populations, strongly linked to adverse health outcomes, and emphasizes the need for improved screening methods to enable effective, targeted care. Currently, seven references remain in burst status (52–58), focusing primarily on guidelines and research related to frailty assessment and nutrition management. Collectively, these studies highlight the essential connection between nutrition and physical health in frail older adults, advocating for early screening and nutritional interventions to improve health outcomes.

Reference clustering analysis provides additional insights into research hotspots and their temporal evolution, offering a comprehensive view of the field’s development (Figure 7C). Foundational clusters, such as cluster #6 (home-living elderly), have evolved into newer clusters like cluster #0 (energy metabolism), cluster #14 (community-dwelling elderly), cluster #5 (geriatric assessment), and cluster #3 (sarcopenia). Recent research directions include cluster #4 (malnutrition), cluster #3 (oral health), cluster #5 (prognosis), cluster #9 (dietary habits), and cluster #8 (cognitive frailty) (Figure 7D). The reference clustering landscape (Figures 7E, F) reveals rapid growth in clusters #3 (oral health) and #4 (malnutrition) in recent years, establishing them as current research hotspots. These trends reflect an increasing emphasis on malnutrition and oral health as key components in frailty prevention and management. Leveraging these insights allows researchers to focus on emerging topics, strengthen collaborative networks, and advance frailty-related research and interventions to better address aging populations’ needs.

### 4.2 Hotspots and frontiers

Bibliometrics’ primary value lies in its capacity to identify research hotspots and emerging trends within a field (28, 59). Keywords, as the essence of scholarly articles, encapsulate the focal points of research. Monitoring the evolution of key terms over time enables the analysis of a field’s development and the identification of new directions and areas of interest (28, 59). In the context of frailty and nutritional status in older adults, current keywords predominantly encompass public health, environmental and occupational health, nutrition and dietetics, geriatrics and gerontology, oncology, pharmacology, surgery, healthcare services, immunology, general and internal medicine, environmental sciences, and microbiology (Figure 8A). These disciplines represent central areas of inquiry in the field.

Analyzing keyword bursts across different time periods reveals the progression of research within this domain. A broad examination of keyword trends from 2005 to 2024 offers insight into the shifts in focus within frailty and nutritional status research for older adults (Figure 8F). Early research concentrated on the impact of environmental factors on frailty risks, particularly weight loss, body composition changes, and functional status among older adults in nursing home settings. During this period, researchers examined the effects of institutional care environments on the nutritional status of frail older adults. The subsequent shift in focus turned toward the intersection of disease and malnutrition, with particular emphasis on health concerns among older women. Key studies during this period examined the effects of chemotherapy on nutrition and quality of life in patients with breast cancer, along with the identification of aging-related malnutrition predictors. More recent studies focus on early detection and management of malnutrition. Common tools include predictive models and the MNA, which help identify at-risk individuals during early stages. Efforts have centered on enhancing nutritional status through supplementation and multi-faceted interventions, assessing the effectiveness of these approaches in delaying frailty progression and improving health outcomes and quality of life for older adults. These trends align with reference clustering analyses (Figures 7C, D).

In summary, research on frailty and malnutrition among older adults has evolved through several phases: care environment studies, health risk assessments, nutritional monitoring, and targeted interventions. These efforts have played a pivotal role in improving the quality of life for older adults. Keywords such as “prediction,” “nutritional assessment MNA,” “intervention,” and “infection” have exhibited persistent bursts from 2022 onward, underscoring their status as key research areas.

#### 4.2.1 The clinical value of the MNA in nutrition screening of older adults

Malnutrition significantly impacts the health and functional status of older adults (61, 62). Studies show that approximately 92.1% of malnourished individuals exhibit signs of frailty, a condition strongly linked to increased all-cause mortality (9, 63). Thus, the accurate assessment of nutritional status in older adults—particularly early identification and intervention for malnutrition—is critical for preventing and delaying frailty. Our analysis reveals that current research focuses on nutritional assessment tools for older adults, with particular attention given to the MNA. Keyword burst analyses underscore “Nutritional Assessment MNA” as a key term in the field, and the keyword timeline from 2005 to 2024 positions “Nutritional Assessment MNA” as a recent addition to cluster #1 (geriatric assessment). Furthermore, co-citation clustering from 2019 to 2024 (Figures 7D, F) highlights cluster #4 (malnutrition) as a primary research focus, reinforcing its status as a current hotspot.

The MNA has been extensively refined and validated. Today, it is recognized worldwide and widely used in community, hospital, and long-term care settings. Many clinical practice guidelines include the MNA as a standard assessment tool (64). The MNA is available in three versions: the full version, the short-form version (MNA-SF), and the self-assessment version. The MNA-SF is particularly effective in clinical practice, demonstrating high sensitivity and specificity, with reported values of 97.6% and 82.8%, respectively, for malnutrition screening in older adults (65–67). To improve its applicability across different populations, the MNA has been adapted in several countries. For instance, Ethiopia adjusted the scoring thresholds, while Thailand replaced BMI with Mindex and Demiquet, enhancing the tool’s sensitivity and specificity (66, 68).

Numerous studies have linked MNA scores to adverse clinical outcomes in older adults. In long-term care settings, MNA scores are positively correlated with functional status, physical performance, and activity levels, while malnourished individuals are more likely to develop frailty (61). Among older adults with dementia, lower MNA scores are strongly associated with higher risks of frailty, depression, and diminished quality of life (odds ratio: 4.76, *p* < 0.01) (69). A study in China found that 46.19% of hospitalized older patients were identified as malnourished or at risk of malnutrition using the MNA-SF (70). Additionally, low MNA-SF scores were significantly linked to prolonged hospital stays, increased mortality, and higher 90-day readmission rates (70). Low MNA scores are also predictive of long-term mortality, with lower scores correlating with significantly increased death risks over a 10-year period (67, 70, 71).

In summary, the MNA is an essential tool for assessing nutritional status and predicting adverse outcomes in older adults. It enables early identification of at-risk individuals, allowing for targeted interventions that improve health outcomes and quality of life (64, 66). However, some limitations in its practical application remain, including variability in sensitivity and specificity across different studies and the time-intensive nature of the assessment process (21, 72). Addressing these challenges could further enhance the effectiveness and efficiency of the MNA in diverse clinical settings.

#### 4.2.2 The application of predictive models in the early identification of malnutrition

Recent advancements in artificial intelligence have facilitated the development of machine learning and big data-driven predictive models, enabling the analysis of complex relationships among multidimensional variables in older adults (73). These models offer significant potential for the early detection and intervention of malnutrition within this population. Keyword burst analysis identified “Prediction” as a prominent term, while timeline analysis revealed “malnutrition risk” as a recent focus within cluster #2 (cognitive frailty), highlighting its increasing relevance in current research.

With the growth of multimodal data and new technologies, combining the MNA with biochemical or imaging assessments has become a key research focus, enhancing the accuracy and comprehensiveness of nutritional evaluations. Studies indicate that machine learning algorithms, including LightGBM, XGBoost, and random forest, achieve an area under the curve (AUC) values above 90% for malnutrition detection in older adults (74). Routine biochemical tests also demonstrate strong predictive value, with an AUC of 0.79, sensitivity of 66.0%, and specificity of 78.1% (52, 75–77). Key biochemical markers—such as low serum albumin, prealbumin, hemoglobin, total cholesterol, and vitamin D levels, as well as elevated C-reactive protein—are critical indicators of malnutrition risk (75–77). These markers provide accurate insights into nutritional status and associated risks. Additionally, machine learning models utilizing facial feature recognition have demonstrated 73.1% accuracy in predicting nutrition risk, offering a non-invasive, cost-effective, and accessible approach for early intervention (78).

Deep learning models, particularly Long Short-Term Memory (LSTM) recurrent neural networks, have shown even greater predictive power. These models, when applied to longitudinal patient data, achieve AUROC values between 0.854 and 0.869, demonstrating their ability to predict malnutrition with high accuracy (79). Models that integrate clinical data, biomarkers, and lifestyle factors have also proven effective in frailty prediction. For example, a multicenter frailty prediction model combining physiological, psychological, and biological variables accurately estimated the 30-day frailty risk in malnourished patients, achieving an AUC of 0.71 (80).

While significant progress has been made in utilizing biomarkers and predictive models for malnutrition detection, challenges remain in their practical application. Variability in algorithm complexity, predictive performance, and model applicability across settings is a key issue, with some models failing external validation tests. Future research should focus on conducting large-scale, multicenter studies to refine existing models, enhancing their generalizability and practical utility. Additionally, exploring multimodal data fusion and incorporating digital health technologies for real-time monitoring and early warning systems will be essential. These efforts will support personalized nutrition management interventions, ultimately improving health outcomes and quality of life for older adults.

#### 4.2.3 The role of nutrition interventions in managing and reversing frailty

In recent years, nutrition interventions have garnered increasing attention as a potential strategy for managing and reversing frailty, signaling a shift from traditional pharmacological and rehabilitation therapies toward nutrition support (15, 16, 81, 82). This study highlights the continued prominence of “intervention” and “infection” as emerging keywords in the 2019–2024 burst analysis. Keyword timeline analysis from 2019 to 2024 reveals that cluster #0 (body composition) is the largest and fastest-growing research cluster, while terms such as intrinsic capacity, fatty acids, and oral frailty appear as significant bursts in VOSviewer analysis (Figure 8D). Additionally, cluster #0 (muscle strength) features recent keywords like sedentary behavior, perspectives, and dietary inflammatory index, while cluster #1 (geriatric assessment) includes ascorbic acid, and cluster #2 (cognitive frailty) emphasizes intrinsic capacity. These trends underscore the ongoing emphasis on nutrition interventions within frailty research.

Malnutrition exacerbates frailty through several mechanisms, including micronutrient deficiencies, cognitive decline, physical weakness, and diminished quality of life (16, 20, 83). Nutrition interventions can slow frailty progression by preserving muscle mass and strength, preventing unintended weight loss, and ensuring adequate intake of essential nutrients and energy (84). Current nutrition strategies encompass dietary supplementation, combined nutrition and physical activity programs, personalized nutrition counseling, and social support (22).

Adequate protein intake (1.2–1.5 g/kg/day) has been shown to improve muscle synthesis, physical function, and mobility, with moderate-quality evidence supporting these benefits (85, 86). However, protein supplementation alone offers limited benefits without concurrent resistance training (87). Antioxidants and anti-inflammatory nutrients are increasingly recognized as potential interventions for mitigating frailty (88). Deficiencies in micronutrients such as vitamin D, B vitamins (folate and B12), albumin, vitamins A and E, omega-3 fatty acids, and antioxidants (e.g., lutein and zeaxanthin) are significantly associated with both physical and cognitive frailty (20, 81, 89, 90). Inadequate intake of minerals like zinc, selenium, copper, magnesium, and potassium—especially zinc—has been linked to heightened frailty and inflammation risks (22, 91). Vitamin C deficiency correlates with a higher likelihood of moderate to severe frailty (92). Conversely, higher dietary and plasma levels of carotenoids (e.g., α-carotene, β-carotene, lutein, lycopene, β-cryptoxanthin) are associated with reduced risks of frailty, suggesting their potential role in prevention (93–95). High-quality dietary patterns, such as the Mediterranean diet, which emphasizes whole grains, vegetables, fruits, nuts, and fish while minimizing ultra-processed foods, are strongly linked to a lower risk of frailty (88, 96, 97). The effectiveness of vitamin D3 and omega-3 fatty acid supplementation in preventing frailty appears to vary depending on baseline nutritional status and individual risk profiles, with emerging evidence suggesting potential benefits in nutritionally vulnerable older adults (98–100).

Emerging evidence indicates that multimodal interventions, combining nutrition with physical activity and psychological support, outperform standalone nutrition strategies in improving frailty, physical function, and mobility (86, 101). A multifactorial intervention combining nutrition education with protein-energy supplementation resulted in a 75% improvement in frailty status and a 58% improvement in physical function (102). Community-based programs, such as the DEFRAIL project, demonstrated frailty reversal through integrated exercise and nutrition interventions (103). Nutrition interventions combined with cognitive and psychological support have proven especially beneficial for older adults with cognitive decline and emotional issues, improving mental health, quality of life, and nutritional status (104, 105). Internet-based multimodal post-discharge programs have improved physical function, appetite, well-being, and quality of life while preventing functional decline (106, 107).

While multimodal nutrition interventions show promise in enhancing physical function and quality of life for frail older adults, the impact of standalone nutrition supplementation remains limited and varies by individual (15, 108). Future research should focus on exploring the causal pathways between nutrition and frailty, identifying the most effective dietary strategies for frailty prevention and treatment, evaluating the long-term efficacy and sustainability of nutrition interventions, and leveraging digital tools for dynamic monitoring of nutritional status to deliver personalized interventions. Advancing these areas will enable nutrition interventions to play a critical role in improving quality of life, reducing healthcare costs, and supporting the health management of aging populations.

#### 4.2.4 From disease-centric to proactive care: mapping real-world changes through bibliometrics

The evolution of research trends observed in this study reflects not only academic interests but also practical transformations in the management of frailty and nutrition in older adults. For instance, the growing prominence of the MNA and predictive models aligns with the increased implementation of routine nutritional risk screening in clinical and community settings, as recommended by the European Society for Clinical Nutrition and Metabolism (ESPEN), the American Geriatrics Society (AGS), and national policies in countries such as Japan, Korea, and China. Similarly, the burst of intervention-related keywords mirrors a paradigm shift from disease-centric to preventive, holistic models of geriatric care. Multimodal nutrition-based interventions are increasingly being integrated into national health plans and long-term care insurance schemes, reflecting a broader policy emphasis on “aging in place” and active aging. Moreover, the rise of machine learning and biomarker-based prediction tools in the literature coincides with healthcare systems’ growing adoption of digital health and precision nutrition platforms. These bibliometric shifts thus provide a window into the changing landscape of elderly care—from reactive interventions to proactive, personalized approaches grounded in data. In sum, the trends identified in our bibliometric analysis not only signify emerging research directions but also echo systemic changes in clinical practice and public health policy, underscoring the translational value of bibliometric methods in capturing shifts in real-world healthcare paradigms.

### 4.3 Limitations

This study applies bibliometric methods to identify research hotspots and trends in frailty and nutritional status among older adults. However, several limitations should be acknowledged. First, the analysis was based solely on the Web of Science database for literature collection. While WoSCC is widely recognized for its comprehensive and high-quality coverage, its inclusion criteria and indexing policies may result in the underrepresentation of relevant studies published in non-English languages or regional journals not covered by the database. As a result, the findings may be biased toward English-language publications and research from higher-income countries (109). To address this, future research could integrate additional databases, such as Scopus, PubMed and CNKI, along with non-traditional sources, including conference proceedings, patent data, and gray literature, to broaden the scope and depth of the analysis. Second, while current bibliometric software offers robust functionality, challenges persist in visualizing complex datasets. Employing artificial intelligence to create dynamic, real-time, and interactive knowledge maps could enhance the depth and flexibility of analyses, enabling more comprehensive exploration within the field.

## 5 Conclusion

This study systematically examined the literature on frailty and nutritional status in older adults through bibliometric analysis, identifying key countries, institutions, authors, and journals driving research in this field. The analysis also mapped international collaboration networks, traced the evolution of the research landscape, and highlighted emerging hotspots and trends. From 2005 to 2024, the volume of publications exhibited a linear growth trajectory (R^2^ = 0.84) and is projected to continue increasing, with the annual publication peak estimated around 2033, reaching approximately 315 publications. These findings underscore the growing importance of this research domain and its sustained appeal to the academic community. The USA and France currently lead in influence, supported by prominent institutions such as INSERM, CIBER, and the Catholic University of the Sacred Heart, which contribute significantly to impactful research. Notable authors include Vellas Bruno, De Vito Francesco, and Marzetti Emanuele, while key journals such as *Nutrients*, *Journal of Nutrition*, *Health & Aging*, and *BMC Geriatrics* serve as primary publication platforms.

Early research focuses on issues related to older adults living at home, with a thematic shift from molecular biology, genetics, and health nursing to clinical medical applications. Current research keywords cluster around disciplines including nutrition and dietetics, geriatrics and gerontology, oncology, and pharmacology. Emerging trends emphasize early malnutrition screening, precision identification of high-risk populations, and the implementation of targeted interventions aimed at preventing and delaying frailty progression. These advancements seek to enhance health outcomes and quality of life for older adults. Keywords such as “prediction,” “nutritional assessment MNA,” “intervention,” and “infection” reflect growing research trends. These findings provide an objective overview of the field, offering valuable insights to inform academic research, policy-making, resource allocation, and the development of future studies in this critical area.

## Data Availability

The original contributions presented in this study are included in this article/Supplementary material, further inquiries can be directed to the corresponding author.
