# Supplementary material for: Predicting the unseen: nutritional interventions as a key to combat frailty
Source: Front Nutr. 2025 Jul 9;12:1575922. doi: 10.3389/fnut.2025.1575922 (PMC12283294; doi:10.3389/fnut.2025.1575922)
Supplement: Supplementary file 1 [file Table_1.docx]

**Supplementary Table S1. The Top10 High Cited Articles on nutrition status in frailty**

| **Rank** | **Title** | **First Author** | **Journal** | **Year** | **Nc** | **Document Type** | **DOI** |
| --- | --- | --- | --- | --- | --- | --- | --- |
| 1 | Gut microbiota composition correlates with diet and health in the elderly | Claesson, Marcus J. | Nature | 2012 | 2573 | Article | 10.1038/nature11319 |
| 2 | International Society of Geriatric Oncology Consensus on Geriatric Assessment in Older Patients With Cancer | Wildiers, Hans | Journal Of Clinical Oncology | 2014 | 1256 | Review | 10.1200/JCO.2013.54.8347 |
| 3 | Through Ageing, and Beyond: Gut Microbiota and Inflammatory Status in Seniors and Centenarians | Biagi, Elena | Plos One | 2010 | 1209 | Article | 10.1371/journal.pone.0010667 |
| 4 | Nutritional, Physical, Cognitive, and Combination Interventions and Frailty Reversal Among Older Adults: A Randomized Controlled Trial | Ng, Tze Pin | American Journal Of Medicine | 2015 | 488 | Article | 10.1016/j.amjmed.2015.06.017 |
| 5 | European Society for Swallowing Disorders European Union Geriatric Medicine Society white paper: oropharyngeal dysphagia as a geriatric syndrome | Baijens, Laura W. J. | Clinical Interventions In Aging | 2016 | 454 | Review | 10.2147/CIA.S107750 |
| 6 | Frailty Syndrome: A Transitional State in a Dynamic Process | Lang, Pierre-Olivier | Gerontology | 2009 | 452 | Article | 10.1159/000211949 |
| 7 | Validity of the Kihon Checklist for assessing frailty status | Satake, Shosuke | Geriatrics & Gerontology International | 2016 | 410 | Article | 10.1111/ggi.12543 |
| 8 | The Asia-Pacific Clinical Practice Guidelines for the Management of Frailty | Dent, Elsa | Journal Of The American Medical Directors Association | 2017 | 420 | Article | 10.1016/j.jamda.2017.04.018 |
| 9 | Risk Factors for Malnutrition in Older Adults: A Systematic Review of the Literature Based on Longitudinal Data | Moreira, Nadia Cristina Favaro | Advances In Nutrition | 2016 | 414 | Review | 10.3945/an.115.011254 |
| 10 | ESPEN guidelines on enteral nutrition:: Geriatrics | Volkert, D. | Clinical Nutrition | 2006 | 409 | Review | 10.1016/j.clnu.2006.01.012 |
